# Admission testing for higher education: A multi-cohort study on the validity of high-fidelity curriculum-sampling tests

**DOI:** 10.1371/journal.pone.0198746

**Published:** 2018-06-11

**Authors:** A. Susan M. Niessen, Rob R. Meijer, Jorge N. Tendeiro

**Affiliations:** Department of Psychometrics and Statistics, Faculty of Behavioral and Social Sciences, University of Groningen, Groningen, the Netherlands; Indiana University, UNITED STATES

## Abstract

We investigated the validity of curriculum-sampling tests for admission to higher education in two studies. Curriculum-sampling tests mimic representative parts of an academic program to predict future academic achievement. In the first study, we investigated the predictive validity of a curriculum-sampling test for first year academic achievement across three cohorts of undergraduate psychology applicants and for academic achievement after three years in one cohort. We also studied the relationship between the test scores and enrollment decisions. In the second study, we examined the cognitive and noncognitive construct saturation of curriculum-sampling tests in a sample of psychology students. The curriculum-sampling tests showed high predictive validity for first year and third year academic achievement, mostly comparable to the predictive validity of high school GPA. In addition, curriculum-sampling test scores showed incremental validity over high school GPA. Applicants who scored low on the curriculum-sampling tests decided not to enroll in the program more often, indicating that curriculum-sampling admission tests may also promote self-selection. Contrary to expectations, the curriculum-sampling tests scores did not show any relationships with cognitive ability, but there were some indications for noncognitive saturation, mostly for perceived test competence. So, curriculum-sampling tests can serve as efficient admission tests that yield high predictive validity. Furthermore, when self-selection or student-program fit are major objectives of admission procedures, curriculum-sampling test may be preferred over or may be used in addition to high school GPA.

## Introduction

Curriculum-sampling tests are increasingly used in admission procedures for higher education across Europe. For example, in Finland, Belgium, the Netherlands, and Austria these test are used across various academic disciplines such as medicine [1–3], psychology [4,5], teacher education [6], economics and business [7], and computer science [8]. The rationale behind these tests is to mimic later behavior that is expected during an academic study. Thus, curriculum samples often mimic representative parts of the academic program that the student is applying to. Often, these samples are small-scale versions of an introductory course of a program, because performance in such courses is a good indicator for later academic performance (e.g., [4,9,10]). An example is studying domain-specific literature or watching video-lectures, followed by an exam.

There are several arguments for using curriculum samples in admission to higher education in addition to or instead of traditional admission criteria used in higher education, such as high school GPA. High school GPA is a good predictor of academic performance in higher education (e.g., [11–14]). However, due to the increasing internationalization and different educational ‘routes’ to higher education [15], these grades are often difficult to compare across applicants. Also, Sackett et al. [16] and Kuncel et al. [17] found that matching the content of the predictor to the criterion was beneficial to the predictive validity.

There are, however, few studies in which the validity of curriculum sampling tests has been investigated. Two types of relevant validity evidence can be distinguished: predictive validity for academic achievement, showing the value of these tests in admission procedures, and construct validity or “construct saturation” (i.e., the degree to which the score variance reflects construct variance [18–20]), which can contribute to *explaining* the predictive validity of curriculum samples. In the first study described in this paper we investigated the predictive validity of curriculum sampling for academic achievement in a psychology program. In the second study, we investigated the construct saturation of curriculum samples.

### Signs, samples, and construct saturation

Most traditional assessments for performance prediction, like cognitive ability tests and personality inventories, are based on a signs approach. Signs are psychological constructs that are theoretically linked to the performance or behavior of interest [21]. In contrast, samples are based on the theory of behavioral consistency: Representative past or current performance is the best predictor for future performance [21,22]. The samples approach originated in the context of personnel selection, where high-fidelity simulations like work sample tests and assessment centers were good predictors of future job-performance [18,23,24]. An explanation for the high predictive validity of sample-based assessments is the ‘point-to-point correspondence’ of the predictor and the criterion [25]. Curriculum-sampling tests are based on the same rationale as work sample tests; they are designed as high-fidelity simulations of (parts of) an academic program.

It is often assumed that sample-based assessments are multifaceted compound measures that are saturated with cognitive- and noncognitive constructs that also underlie performance on the criterion task [22,26]. The construct saturation an assessment represents the degree to which score variance reflects variance on different constructs. The concept of construct saturation may seem in conflict with the underlying idea of a samples approach, since they are explicitly not designed to measure distinct constructs. However, construct saturation studies can help in explaining the predictive validity of sample-based assessments through investigating what scores on sample-based assessments represent in terms of psychological constructs [20]. In addition, construct saturation can affect predictive validity and the size of sub-group score differences of test scores [18,19,27]. Especially noncognitive saturation may provide great benefits in high-stakes assessment. Noncognitive constructs like personality traits, self-efficacy, and self-regulation are good predictors of future job-performance and academic performance, and show incremental validity over cognitive abilities [28]. However, they are difficult to measure validly in high-stakes assessment due to faking [29,30]. A performance-based assessment method that is able to tap into noncognitive traits and skills may provide a solution to that problem.

### Curriculum sampling: Existing research

Curriculum-sampling tests often require studying domain-specific literature or–lectures, followed by an exam, but this approach can take many different forms, depending on the curriculum the test is designed for. The approach may also involve the preparation of a demonstration lesson in the case of teacher education [6], in which internships within schools make up a large proportion of the curriculum, a massive online open course (MOOC) for admission to a computer science program, in which applicants successfully have to complete programming assignments from the first academic year [8], or a ‘virtual semester’ in medical school, followed by an exam [3].

Most studies on curriculum sampling compared the academic performance of students admitted through curriculum-sampling procedures with students admitted via other methods. Results showed that students admitted through a curriculum-sampling procedure earned higher grades, progressed through their studies faster, and dropped out less often compared to students admitted via lottery [1,5] or via traditional entrance tests or matriculation exam grades [8]. However, participation in the curriculum-sampling admission procedures was voluntary in these studies, and admission could also be achieved through other procedures. Thus, these differences may also be caused by, for example, motivation; highly motivated applicants may have chosen to participate in the curriculum-sampling procedure that requires effort, whereas less motivated applicants may have chosen an alternative route [31].

Reibnegger et al. [3] compared the dropout rate and time to completion of the first part of medical school for three cohorts of students admitted through open admission, a ‘virtual semester’ in medicine followed by a two-day examination, or secondary school-level knowledge exams about relevant subjects. The best results were found for the cohort admitted through the ‘virtual semester’. However, the selection ratio was also the lowest for that cohort, which may have influenced the results.

Lievens and Coetsier [2] examined the predictive validity of two curriculum-sampling tests for medical school for first year GPA, one using a video-based lecture (*r* = .20) and one using written medical material (*r* = .21). However, these tests had relatively low reliability (α = .55 and .56, respectively). Their study is, to our knowledge, also the only study that investigated the construct saturation of curriculum sampling tests. They found moderate relationships between the curriculum-sampling exams scores and scores on a cognitive ability test (*r* = .30 and *r* = .31, respectively), indicating at least some cognitive ability saturation, but they did not find relationships with scores on the Big Five personality scales. In addition, Niessen et al. [4] found that the predictive validity of a curriculum-sampling test for undergraduate psychology applicants was *r* = .49 for first year GPA, *r* = .39 for credits obtained in the first year, and *r* = -.32 for dropping out of the program in the first year. Furthermore, the curriculum-sampling test scores were related to subsequent self-chosen enrollment, indicating that it may serve as a self-selection tool. Booij and van Klaveren [7] found similar results based on an experiment in which applicants to an economics- and business program were randomly assigned to a non-binding placement procedure consisting of an intake interview or a curriculum-sampling day without a formal exam or assignment. Compared to the interview condition, fewer students from the curriculum-sampling condition enrolled in the program and more students passed the first year. Enrolling students who participated in the curriculum-sampling procedure also reported that the program met their expectations more often than enrollees from the interview condition.

### Aim of the current article

The few existing predictive validity studies [2,4] only used single cohorts and only used first year academic achievement as a criterion measure. To our knowledge, there are no predictive validity studies using outcomes such as graduation rates and long-term college GPA. Furthermore, there is only one study [2] in which the construct saturation of curriculum-sampling tests was investigated. To address these shortcomings in the literature, we conducted two studies. The aim of the first study was to investigate the predictive validity of curriculum-sampling tests for academic performance in a psychology program at a Dutch university. We studied predictive validity in different cohorts, using not only first year GPA but also third year GPA and bachelor-degree attainment as criterion measures, thus extending the Niessen et al. [4] study. The aim of the second study was to investigate the construct saturation of curriculum-sampling tests by gaining more insight into how these test scores are related to psychological constructs, in order to explain their predictive validity.

#### The present studies

The academic outcomes in Study 1 were academic performance, study progress, and college retention. In addition, we studied (1) the incremental validity of the curriculum-sampling scores over high school GPA, (2) the predictive- and incremental validity of curriculum-sampling tests for achievement in specific courses, over specific skills tests designed to predict performance in those courses [16], and (3) the relationship between curriculum-sampling tests scores and self-chosen enrollment decisions. We studied two types of curriculum-sampling tests: A literature-based test and a video-lecture test. Given the correspondence between criterion and the predictors, we expected to replicate the high predictive validity results from Niessen et al. [4] for first year academic achievement and we expected somewhat lower predictive validity for later academic achievement. In addition, we expected that the skills tests would predict performance in courses they were designed to predict (the math test for statistics courses and the English test for theoretical courses), and that the curriculum-sampling tests would predict performance in both types of courses, but that the correlation with statistics course performance would be lower compared to the math test.

In the second study we investigated the hypothesis that the curriculum-sampling tests are saturated with both cognitive- and noncognitive constructs. Assuming that the curriculum-sampling test scores represent a ‘sample’ of future academic performance, we expected that the curriculum-sampling test scores would be saturated with variables that also predict academic performance, such as cognitive ability [32,33] and several noncognitive constructs and behavioral tendencies [28,34]. To investigate this hypothesis, we studied if and to what extent the scores on the curriculum-sampling test can be explained by cognitive ability, conscientiousness, procrastination tendencies, study-related cognitions, and study strategies [28,34].

## Study 1: Predictive validity

### Method

#### Procedure

All applicants to an undergraduate psychology program at a Dutch university were required to participate in an admission procedure. In 2013 and 2014, the admission procedure consisted of the administration of a literature-based curriculum-sampling test and two skills tests: A math test and an English reading comprehension test. In 2015, a math test and two curriculum-sampling tests (a literature-based test and a video-lecture test) were administered. Administration time for each test was 45 minutes, with15-minute breaks in between. Each test score was the sum of the number of items answered correctly. Applicants were ranked based on a composite score with different weights for the individual tests in each cohort. The highest weight was always assigned to the literature-based curriculum-sampling test. All applicants received feedback after a few weeks, including their scores on each test and their rank. In addition, the lowest ranking applicants (20% in 2013 and 15% in 2014 and 2015) received a phone call to discuss their results with an advice to rethink their application. However, the selection committee did not reject applicants because the number of applicants willing to enroll did not exceed the number of available places. The applicants did not know this beforehand, thus the applicants perceived the admission procedure as high stakes. The study program and the procedure could be followed in English or in Dutch. Applicants to the English program were mostly international students.

#### Curriculum-sampling tests

The literature-based curriculum-sampling test was used in each cohort, and was designed to mimic the first-year course *Introduction to Psychology*. The applicants were instructed to study two chapters of the book used in that course. The second curriculum-sampling test, only administered in 2015, required applicants to watch a twenty-minute video lecture on the topic *Psychology and the Brain*. A lecturer who taught a related course in the first year provided the lecture. At the selection day, the applicants completed multiple-choice exams about the material. The exams were similar to the exams administered in the first year of the program and were designed by faculty members who taught in the first year. The first curriculum-sampling test consisted of 40 items in 2013 and 2014, and of 39 items in 2015. The second curriculum-sampling test consisted of 25 items. The exams consisted of different items each year. Cronbach’s alpha for each test is displayed in S1 Table.

#### Skills tests

The English test was included in the procedure because most of the study material is in English, also in the Dutch-taught program, and the math test was included because statistics courses are a substantial part of the program. The English test consisted of 20 multiple choice items on the meaning of different English texts. The math test consisted of 30 multiple choice items in 2013 and 2014, and 27 multiple choice items in 2015, testing high-school level math knowledge. The applicants did not receive specific material to prepare for these tests, but example items were provided for the math test. The tests consisted of different items each year.

#### High school performance

High school grades of enrolled students who completed the highest level of Dutch secondary education (pre-university education, in Dutch: vwo) were collected through the university administration. The grades were not part of the admission procedure. The mean high school grade (HSGPA) was the mean of the final grades in all high school courses, except courses that only resulted in a pass/fail grade. For most courses, 50% of the final course grade was based on a national final exam. The other 50% consisted of the grades obtained in the last three years of secondary education.

#### Academic achievement

Outcomes on academic achievement were collected through the university administration. For all cohorts, the grade on the first course, the mean grade obtained in the first year (FYGPA, representing academic performance), the number of credits obtained in the first year (representing study progress), and records of dropout in the first year (representing retention) were obtained. For the 2013 cohort we also collected the mean grade obtained after three years (TYGPA, representing academic performance) and bachelor degree attainment after three years (representing study progress). The bachelor program can be completed in three years. All grades were on a scale of 1 to 10, with 10 being the highest grade and a six or higher representing a pass. Mean grades were computed for each student using the highest obtained grade for each course (including resits). Courses only resulting in a pass/fail decision were not taken into account. Credit was granted after a course was passed; most courses earned five credit points, with a maximum of 60 credits per year. The first and second year courses were mostly the same for all students; the third year consisted largely of elective courses. Since the skills tests were designed to predict performance in particular courses, we also computed a composite for statistics courses (SGPA) and theoretical courses (all courses that required studying literature and completing an exam about psychological theories; TGPA) in the first year. The SGPA is the mean of the final grades on all statistics courses and the TGPA is the mean final grades on courses about psychological theory. In addition, we also obtained information on whether students chose to enroll after participating in the admission procedure. Because we only used data available at the university, there were no manipulations in this study, and no identifiable information was presented, informed consent was not obtained. This was in line with the university’s privacy policy. This study was approved by and in accordance with the rules of the Ethical Committee Psychology from the University of Groningen [35].

#### Applicants

The 2013 cohort (this is the same cohort used in Niessen et al.[4]) consisted of 851 applicants, of whom 652 (77%) enrolled in the program, and 638 participated in at least one course. For enrollees the mean age was 20 (*SD* = 2.0), 69% was female, 46% were Dutch, 42% were German, 9% had another European nationality, 3% had a non-European nationality, and 57% followed the program in English. A high school GPA obtained at the highest level of Dutch secondary education was available for 201 enrollees. Third year academic performance was available for 492 students, the others dropped out of the program in the first or second year. A high school GPA was available for 159 of these students.

The 2014 cohort consisted of 823 applicants, of whom 650 enrolled in the program (79%) and 635 participated in at least one course. For the enrollees the mean age was 20 (*SD* = 2.1), 66% was female, 44% were Dutch, 46% were German, 7% had another European nationality, 3% had a non-European nationality, and 59% followed the program in English. The high school GPA obtained at the highest level of Dutch secondary education was available for 217 enrollees.

The 2015 cohort consisted of 654 applicants, of whom 541 (83%) enrolled in the program, and 531 participated in at least one course. For enrollees the mean age was 20 (*SD* = 2.0), 70% was female, 43% were Dutch, 46% were German, 9% had another European nationality, 2% had a non-European nationality, and 62% followed the program in English. The high school GPA obtained at the highest level of Dutch secondary education was available for 188 enrollees.

#### Analyses

To assess the predictive validity of the curriculum-sampling tests, correlations were computed for each cohort between the different predictor scores and FYGPA, obtained credits in the first year, dropout, TYGPA, and degree attainment after three years. Because the literature-based curriculum-sampling tests was designed to mimic the first course, and because the first course was previously found to be a good predictor of subsequent first year performance [4,10], correlations between the curriculum-sampling test scores and the first course grade and correlations between the first course grade and subsequent first year performance were also computed. For these analyses, results from the first course were excluded from the FYGPA and the number of obtained credits. In addition, to compare the predictive validity of the curriculum-sampling tests to the predictive validity of HSGPA, correlations between HSGPA and the academic performance outcomes were also computed. In addition, the relationship between the admission test scores and enrollment was studied by computing point-biserial correlations between the scores on the admission tests and enrollment. Low-ranking applicants were contacted by phone and were encouraged to reconsider their application. To further investigate the relationships between enrollment and the scores on the admission tests, logistic regression analyses controlling for receiving a phone call were conducted in each cohort, with the admission test scores as independent variables and enrollment as the dependent variable. To ease interpretation of the odds ratios, the admission test scores were standardized first.

#### Corrections for range restriction

Although applicants were not rejected by the admission committee, indirect range restriction occurred due to self-selection through self-chosen non-enrollment for first year results, and through dropout in earlier years of the program for third year results, which may result in underestimation of operational validities. Therefore, the individual correlations (*r*) were corrected for indirect range restriction (IRR) using the Case IV method [36] resulting in an estimate of the true score correlation (ρ) corrected for unreliability in the predictor and the criterion, and for IRR, and the operational validity (*r*_*c*_), only corrected for IRR and unreliability in the criterion variable. Corrections for criterion unreliability were only made for GPA criterion variables, and not for obtained credits and drop out. The corrected correlations were computed using the *selection* package in R [37]. In the discussion of the results we focus on the operational validities [38]. Statistical significance (α = .05) of individual correlations was determined before corrections were applied. The correlations were aggregated across cohorts when applicable (resulting in $\bar{r},\;\bar{\rho }$, and ${\bar{r}}_{c}$).

Because the number of cohorts was small and the admission procedures and samples were very similar, the validity estimates were aggregated ($\bar{r},\;\bar{\rho }$, and ${\bar{r}}_{c}$) applying a fixed effects model, using the *metafor* package in R [39]. It was not possible to correct the correlations between first year academic results and HSGPA and the first course grade for IRR, since only data of enrolled students were available for these variables. Therefore, these correlations were only corrected for predictor- and criterion unreliability $\left(\bar{\rho }\right)$, and for criterion unreliability $\left({\bar{r}}_{c}\right)$. For third year results, the correlations for the first course grade and high school GPA could be corrected for range restriction due to dropout in earlier years. The reliability of the first course grade was only known for the 2013 sample (α = .74) and was assumed constant across cohorts.

In addition, the incremental validity of the literature-based curriculum-sampling test over HSGPA was studied based on the observed $\left(\bar{r}\right)$ and corrected $\left({\bar{r}}_{c}\right)$ aggregated correlations, including an IRR correction on the correlation between HSGPA and the curriculum-sampling test scores. We conducted these analyses using the full samples, and using only data from students that had a high school GPA at the highest level of Dutch secondary education. Furthermore, the skills tests (math and English reading comprehension) were included in the admission procedure to predict performance in first year statistics courses and theoretical courses, respectively. We studied the predictive validity for these courses by computing correlations between the scores on the admission tests and the mean grade on these two types of courses in the first year, and the incremental validity of the skills tests over the literature-based curriculum-sampling test. The corrections and aggregation procedures described above were also applied to these analyses.

#### Estimating GPA reliability

Reliability estimates for GPA variables were obtained in the same way as described in Bacon and Bean [9]. First, we computed intraclass correlations (ICC’s) between the grades that were used to compute each GPA variable, using the mean squares resulting from ANOVA’s with grade as the DV and student as the IV. Next, the Spearman-Brown prophecy formula was applied to the ICC’s using the mean number of grades in each GPA variable. The resulting reliability estimates are shown in S2 Table and were in line with previous results on the reliability of college GPA [9,40]. The same procedure was used to compute the reliability of high school GPA, shown in S1 Table.

### Results

#### Short-term predictive validity

S1 and S2 Tables contain all descriptive statistics and S3 Table shows the observed correlations between the predictors and the first year academic outcomes in each cohort. Table 1 shows the aggregated observed, true, and operational validities of each predictor measure for the first year academic performance outcomes.

**Table 1:** Correlations between predictors and first year academic outcomes aggregated across cohorts.

Predictor	FYGPA			FYECT			FY dropout^a^			Enrollment^a^	
	$\bar{r}$	$\bar{\rho }$	${\bar{r}}_{c}$	$\bar{r}$	$\bar{\rho }$	${\bar{r}}_{c}$	$\bar{r}$	$\bar{\rho }$	${\bar{r}}_{c}$	$\bar{r}$	$\bar{\rho }$
Cur. 1	.46 [.43,.50]	.63 [.58,.68]	.56 [.52,.61]	.36 [.32,.40]	.47 [.42,.52]	.42 [.37,.47]	-.27 [-.31,-.23]	-.36 [-.42,-.30]	-.32 [-.37,-.27]	.25 [.22,.29]	.29 [.24,.33]
Cur. 2^b^	.29 [.21,.37]	.43 [.29,.51]	.36 [.25,.43]	.25 [.17,.33]	.35 [.24,.45]	.29 [.20,.38]	-.13 [-.21,-.05]	-.18 [-.29,-.07]	-.15 [-.25,-.06]	.18 [.11,.25]	.21 [.13,.30]
Math	.25 [.21,.30]	.32 [.27,.38]	.28 [.23,.33]	.18 [.14,.22]	.21 [.16,.27]	.19 [.14,.23]	-.13 [-.17,-.08]	-.15 [-.21,-.10]	-.13 [-.18,-.09]	.09 [.05,.13]	.10 [.06,.15]
English	.17 [.12,.23]	.26 [.18,.34]	.21 [.15,.27]	.12 [.07,.17]	.17 [.10,.24]	.13 [.08,.19]	-.10 [-.15,-.04]	-.13 [-.21,-.06]	-.11 [-.17,-.05]	.15 [.10,.20]	.19 [.31,.24]
HSGPA^c^	.47 [.41,.53]	.60 [.53,.67]	.50 [.43,.57]	.28 [.20,.35]	.33 [.25,.41]		-.20 [-.27,-.12]	-.24 [-.32,-.16]			
FCG^c^^,^^d^	.72 [.69,.74]	.89 [.86,.92]	.76 [.74,.79]	.61 [.58,.64]	.70 [.68,.74]		-.43 [-.47,-.39]	-.51 [-.55,-.47]			

Cur. 1 = curriculum-sampling test based on literature, Cur. 2 = curriculum-sampling test based on a video lecture, Math = math test, English = English reading comprehension test, HSGPA = high school mean grade, FCG = first course grade, FYGPA = first year mean grade, FYECT = first year credits, FY dropout = first year dropout, Enrollment = first year enrollment, $\bar{r}$ = the aggregated correlation across cohorts, $\bar{\rho }$ = the aggregated true score correlation (corrected for unreliability and indirect range restriction), ${\bar{r}}_{c}$ = the aggregated operational correlation across cohorts (corrected for indirect range restriction).

^a^ Point-biserial correlations.

^b^ Based the 2015 cohort.

^c^ These correlations could not be corrected for IRR, just for unreliability.

^d^ For these correlations, results on the first course were not included in the calculation of FYGPA and credits. 95% confidence intervals are in brackets. All correlations were statistically significant with *p* < .05.

#### Curriculum samples as predictors

The validity of the literature-based curriculum-sampling test was consistent across cohorts and the aggregated operational validity was high for first year academic performance in terms of GPA $\left({\bar{r}}_{c}=.56\right)$ and moderate for obtained credits $\left({\bar{r}}_{c}=.42\right)$ and for dropout in the first year $\left({\bar{r}}_{c}=-.32\right)$. The video-lecture test (only administered in 2015) showed moderate predictive validity for FYGPA (*r*_*c*_ = -.36) and obtained credits (*r*_*c*_ = .29), and a small negative correlation with dropout (*r*_*c*_ = -.15). In the entire applicant sample, the correlation between the scores on both curriculum-sampling tests equaled *r* = .51. In addition, the video-lecture test showed very small incremental validity for predicting FYGPA over the literature-based curriculum-sampling test (Δ*R*^2^ = .01, *R*^2^ = .20, Δ*F*_*(1*, *528)*_ = 5.69, *p* = .02, and based on the corrected correlations, Δ*R*^2^_c_ = .01, *R*^2^_c_ = .29).

#### Specific skills tests and grades as predictors

The operational validities of the math and English skills tests were less consistent across cohorts (S3 Table) and the aggregated operational validities were moderate to small for all outcome measures. The data needed to check for and, if needed, correct for range restriction in HSGPA were not available, so we only computed correlations corrected for unreliability with the academic outcomes.

High school GPA showed high predictive validity for FYGPA $\left({\bar{r}}_{c}=.50\right)$, and moderate predictive validity for the obtained number of credits $\left(\bar{r}=.28\right)$ and dropout $\left(\bar{r}=-.20\right)$. The first course grade showed very high predictive validity for subsequent performance in the first year, with ${\bar{r}}_{c}=.76$ for FYGPA, $\bar{r}=.61$ for obtained credits, and $\bar{r}=-.43$ for dropout. These correlations were substantially higher than those for the literature-based curriculum-sampling test, which was modeled after this first course. The literature-based curriculum-sampling test showed an aggregated correlation with the first course grade of $\bar{r}=.49$ (${\bar{r}}_{c}=.56$ after correction for IRR).

#### Long-term predictive validity

Table 2 shows observed and corrected correlations between the predictors and academic performance after three years (only studied for the 2013 cohort). The literature-based curriculum-sampling test showed a high operational validity of *r*_*c*_ = .57 for third year GPA, and a moderate operational validity of *r*_*c*_ = .32 with bachelor’s degree attainment in three years. The math skills test showed small validities (*r*_*c*_ = .28 for TYGPA and *r*_*c*_ = .19 for TYBA), and the English test showed small validity for TYGPA of *r*_*c*_ = .18, and small, non-significant validity for TYBA (*r*_*c*_ = .06). High school GPA had a high correlation with TYGPA of *r*_*c*_ = .65 and a moderate correlation with TYBA of *r*_*c*_ = .31. Lastly, the first course grade on *Introduction to Psychology* obtained in the first year showed large correlations with TYGPA (*r*_*c*_ = .73) and with TYBA (*r*_c_ = .48). Thus, the curriculum-sampling test scores, high school GPA, and the first course grade were good predictors of academic performance after three years of studying in the Psychology program.

**Table 2:** Correlations between predictors and third year academic outcomes.

Predictor	TYGPA			TYBA^a^		
	*r*	ρ	*r*_*c*_	*r*	Ρ	*r*_*c*_
Cur. 1	.38* [.30,.45]	.64 [53,.72]	.57 [.47,.65]	.20* [.11,.28]	.35 [.20,.48]	.32 [.18,.43]
Math	.24* [.16,.32]	.32 [.22,.43]	.28 [.19,.37]	.17* [.08,.25]	.22 [.10,.32]	.19 [.09,.28]
English	.17* [.08,.25]	.21 [.10,.31]	.18 [.08,.26]	.06 [-.03,.15]	.07 [-.04,.18]	.06 [-.03,.15]
HSGPA^b^	.61 [.50,.70]	.76 [.63,.87]	.65 [.54,.74]	.30* [.15,.44]	.37 [.18,.53]	.31 [.16,.46]
FCG^b^	.62 [.56,.67]	.85 [.80,.89]	.73 [.69,.77]	.37* [.10,.47]	.56 [.46,.65]	.48 [.39,.56]

Cur. 1 = curriculum-sampling test based on literature, Math = math test, English = English reading comprehension test, HSGPA = high school mean grade, FCG = first course grade, TYGPA = third year mean grade, TYBA = third year Bachelor’s degree attainment, *r* = uncorrected correlation, ρ = the aggregated true score correlation (corrected for unreliability and indirect range restriction), *r*_*c*_ = correlation corrected for indirect range restriction.

^a^ Point-biserial correlations.

^b^ These correlations were only corrected for IRR due to drop out after starting the program. 95% confidence intervals are in brackets.

* *p* < .05

#### Incremental validity

The incremental validity of the literature-based curriculum-sampling tests over high school GPA was computed based on the aggregated correlations across cohorts, both observed and operational. These analyses were conducted using data based on the entire samples (Table 3), and on the subsets of applicants who had a high school GPA data (S1 Appendix). The correlations between the curriculum-sampling test scores and academic achievement were similar or slightly lower based on these subsets, compared to the results presented in Table 1 (see S1 Appendix). When the analyses were conducted for each cohort separately (results not shown but available upon request), the incremental validity of the literature-based curriculum-sampling test over HSGPA was statistically significant in each cohort and for each criterion. The aggregated correlation between the curriculum-sampling test score and high school GPA was $\bar{r}=.49$ and ${\bar{r}}_{c}=.55$. The curriculum-sampling test showed a substantial increase in explained variance over high school GPA for predicting FYGPA $\left(\Delta {\bar{R}}_{c}^{2}=.12\right)$, and for predicting the number of obtained credits $\left(\Delta {\bar{R}}_{c}^{2}=.10\right)$. Together, high school GPA and the curriculum-sampling test scores explained a large percentage of the variance in FYGPA $\left({\bar{R}}_{c}^{2}=.37\right)$ and in obtained credits in the first year $\left({\bar{R}}_{c}^{2}=.18\right)$. For predicting TYGPA, the curriculum-sampling test and high school GPA combined explained 49% of the variance, but the incremental validity of the curriculum-sampling test over high school GPA was modest $\left(\Delta {\bar{R}}_{c}^{2}=.06\right)$. The results based on the subsets of applicants with a high school GPA were similar, with slightly lower incremental validity over high school GPA for FYGPA and FYECT, and slightly higher incremental validity over TYGPA (see S1 Appendix).

**Table 3:** Incremental validity of the literature-based curriculum-sampling test over high school GPA.

Data	FYGPA			FYECT			TYGPA^a^		
	$\bar{R}$	${\bar{R}}^{2}$	$\Delta {\bar{R}}^{2}$	$\bar{R}$	${\bar{R}}^{2}$	$\Delta {\bar{R}}^{2}$	*R*	*R*^2^	*ΔR*^2^
Observed	.54	.29	.07	.37	.14	.07	.62	.38	.01
Corrected	.60	.37	.12	.42	.18	.10	.70	.49	.06

FYGPA = first year mean grade, FYECT = first year credits, TYGPA = third year mean grade, $\bar{R}$ = aggregated multiple correlation, ${\bar{R}}^{2}$ = aggregated variance explained based on HSGPA and the curriculum-sampling test scores, $\Delta {\bar{R}}^{2}$ = aggregated increase in explained variance based on curriculum-sampling test scores over HSGPA. Observed = based on observed correlations, Corrected = based on operational correlations (corrected for IRR).

^a^ Based on the 2013 cohort.

#### Specific course achievement

The predictive validity of the admission tests for specific course achievement in the first year is shown in Table 4. The aggregated correlation between the mean grade on the statistics courses (SGPA) and the mean grade on theoretical courses (TGPA) was $\bar{r}=.66$. As expected, the scores on the English test only showed a small correlation with performance in the statistics courses $\left({\bar{r}}_{c}=.16\right)$. The curriculum-sampling tests and the math test predicted performance in statistics course equally well (around ${\bar{r}}_{c}=.50$). Their scores combined accounted for a large percentage of the variance in statistics GPA $\left({\bar{R}}_{c}=.38\right)$, with an incremental validity of the math test over the curriculum-sampling test of $\left(\Delta {\bar{R}}_{c}^{2}=.12\right)$. The curriculum-sampling tests were the strongest predictors of performance in theoretical courses, with ${\bar{r}}_{c}=.57$ for the literature-based test and ${\bar{r}}_{c}=.33$ for the video-lecture test. The English test and the math test showed small correlations (${\bar{r}}_{c}=.20$ and ${\bar{r}}_{c}=.21$) with theoretical course performance. The scores on the English test and the literature-based curriculum-sampling test combined accounted for a large percentage of the variance in theoretical GPA $\left({\bar{R}}_{c}=.33\right)$ and with an incremental validity of the English test over the curriculum-sampling test of $\Delta {\bar{R}}_{c}^{2}<.01$. The results based on the observed, uncorrected correlations are described in S2 Appendix.

**Table 4:** Predictive validity for specific course achievement in the first year.

Predictor	SGPA			TGPA		
	$\bar{r}$	$\bar{\rho }$	${\bar{r}}_{c}$	$\bar{r}$	$\bar{\rho }$	${\bar{r}}_{c}$
Cur. 1	.36 [.32,.40]	.57 [.50,.63]	.51 [.45,.57]	.46 [.43,.50]	.63 [.58,.68]	.57 [.52,.61]
Cur. 2^a^	.33 [.25,.40]	.56 [.43,.66]	.47 [.36,.56]	.26 [.18,.34]	.39 [.27,.50]	.33 [.23,.43]
Math	.37 [.33,.41]	.54 [.48,.60]	.47 [.42,.52]	.19 [.14,.23]	.24 [.18,.29]	.21 [.16,.26]
English	.13 [.07,.18]	.20 [.11,.30]	.16 [.09,.24]	.17 [.12,.22]	.26 [.18,.33]	.20 [.14,.26]

Cur. 1 = curriculum-sampling test based on literature, Cur. 2 = curriculum-sampling test based on a video lecture, Math = math test, English = English reading comprehension test, SGPA = statistics courses GPA, TGPA = theoretical courses GPA, $\bar{r}$ = the aggregated correlation across cohorts, $\bar{\rho }$ = the aggregated true score correlation (corrected for unreliability and indirect range restriction), ${\bar{r}}_{c}$ = the aggregated operational correlation across cohorts (corrected for indirect range restriction). 95% confidence intervals are in brackets.

^a^ Based on the 2015 cohort. All correlations were statistically significant with *p* < .05.

#### Enrollment

The aggregated operational and true score correlations between the admission test scores and enrollment are shown in Table 1, and the observed correlations per cohort are in S3 Table. The aggregated operational correlation between the scores on the literature-based curriculum-sampling tests and enrollment was $\bar{r}=.25$; for the video-lecture test this was *r* = .18. The math test scores $\left(\bar{r}=.09\right)$ and the English test scores $\left(\bar{r}=.15\right)$ showed small correlations with enrollment. So, the admission test scores showed small relationships with enrollment, with the largest correlation for the literature-based curriculum-sampling test.

To assess the relationship between the scores on the admission tests and enrollment further, enrollment was regressed on the admission test scores, controlling for receiving a discouraging phone call. To ease interpretation of the odds ratios, the admission test scores were standardized first. The results of the logistic regression are shown in Table 5. In each cohort, the score on the literature-based curriculum-sampling test was significantly related to enrollment, with higher scores associated to higher odds for enrollment. With each standard deviation unit increase in the test score, the odds of enrollment increased with *e*^*B*^ = 1.33, *e*^*B*^ = 2.04, and *e*^*B*^ = 1.55 in 2013, 2014, and 2015, respectively. In the 2013 and 2014 cohorts, the curriculum-sampling test score was the only significant predictor of enrollment. The video-based curriculum-sampling test score and the math test score also showed statistically significant relationships with enrollment in the 2015 cohort (*e*^*B*^ = 1.34 and *e*^*B*^ = 0.78). Notably, a higher score in the math test was associated with lower odds of enrollment.

**Table 5:** Logistic regression results for predicting enrollment based on admission test scores.

Variable	2013^a^			2014			2015		
	*e*^*B*^	95% CI	Wald	*e*^*B*^	95% CI	Wald	*e*^*B*^	95% CI	Wald
Phone call	1.70	0.97, 2.99	3.38	1.09	0.59, 2.02	0.79	1.09	0.49, 2.42	0.04
Cur. 1	1.33	1.09, 1.63	7.67*	2.04	1.64, 2.53	40.83*	1.55	1.15, 2.07	8.42*
Cur. 2							1.34	1.05, 1.71	5.51*
Math	1.14	0.93, 1.40	1.67	1.04	0.84, 1.30	0.13	0.78	0.61, 0.98	4.43*
English	1.03	0.85, 1.24	0.06	1.11	0.91, 1.36	1.15			
Model *X*^*2*^			46.44*			81.28*			39.10*

Cur. 1 = curriculum-sampling test based on literature, Cur. 2 = curriculum-sampling test based on a video lecture, Math = math test, English = English reading comprehension test. *e*^*B*^ = odds ratio.

^a^ These results (using unstandardized data) were also shown in Niessen et al. [4]

* *p* < .05.

### Discussion

The results showed that the predictive validity of the literature-based curriculum-sampling test for first year academic achievement was consistent across cohorts, with high correlations with FYGPA and moderate correlations with the number of obtained credits and dropout. These results replicated the results obtained by Niessen et al. [4]. However, the validity of the curriculum-sampling test was lower than the predictive validity of the grade in the course that it was designed to mimic; this course grade showed very large correlations with later academic achievement. In addition, the video-lecture based curriculum-sampling test showed moderate predictive validity and little incremental validity over the literature-based test. Notably, the operational predictive validity of the literature-based curriculum-sampling test for academic performance after three years of bachelor courses was still high. The predictive validity of the curriculum-sampling test was mostly comparable to or slightly higher than the predictive validity of high school GPA; in comparison, high school GPA was a better predictor for third year GPA. Furthermore, whereas the literature-based curriculum-sampling test scores and high school GPA were strongly related, the unique explanatory power of the curriculum test scores over high school GPA was substantial.

For the prediction of specific course achievement, our expectations were partially confirmed. The math test scores did not predict statistical course performance better than the curriculum-sampling tests, but they did show some incremental validity over the curriculum-sampling test. The English test scores did not add incremental validity over the curriculum-sampling test for predicting theoretical course performance. A possible explanation may be that English reading comprehension was also implicitly assessed by the curriculum-sampling test, as the material to be studied was in English. Finally, in the regression analyses, relationships between test scores and enrollment decisions were only found for the curriculum-sampling test scores, indicating that this relationship may be an advantage of representative sample-based admission tests in particular.

## Study 2: Construct saturation

### Method

#### Participants and procedure

The sample consisted of 104 first year students who enrolled in the Dutch-taught psychology program in 2015 and participated in the study as part of a course on research methods, for which they had to participate in a number of studies of their own choice. The participants were a little younger than the non-participants in this cohort (the mean age was 19 and 20, respectively) and consisted of a similar percentage of females (71% and 70%, respectively). All participants had a Dutch nationality. In addition, compared to the non-participants, the participants scored slightly lower on the literature-based curriculum-sampling test (*t*_(539)_ = -2.16, *p* = .03, *d* = -0.22), slightly higher on the video lecture-based curriculum-sampling test (*t*_(539)_ = 2.73, *p* < .01, *d* = 0.32), and there were no significant differences in FYGPA (*t*_(529)_ = 0.60, *p* = .55, *d* = 0.08).

The data were collected in two sessions scheduled three days apart. In the first session, participants completed self-report questionnaires about their personality, procrastination tendencies, study skills, and study habits. In the second session they completed a cognitive ability test that took about an hour to complete. All measures were administered on a computer in the Dutch language. Both sessions were proctored and took place in a research lab at the university, with several participants completing the measures simultaneously. The participants received their scores on the cognitive test immediately after completion to encourage effortful participation. All participants provided written informed consent and provided permission to link the data to their admission test scores and academic performance. This study was approved by and in accordance with the rules of the Ethical Committee Psychology from the University of Groningen [35]. Participation took place between October 2015 and April 2016.

#### Cognitive ability

Cognitive ability was measured by the Q1000 Capaciteiten Hoog (in Dutch [41]), developed for personnel selection and career development purposes for adults who followed upper higher education. The test consisted of seven subscales across three domains: Analogies, syllogisms, and vocabulary for the verbal domain; digit series and sums for the numerical domain; and matrices and cubes for the figural domain. The test also yields a higher-order general cognitive ability score consisting of a combination of the three domain scores. The test showed convergent validity with other measures of cognitive ability (the Dutch version of the General Aptitude Test Battery and Raven’s Progressive Matrices [41]), and the Dutch Committee on Tests and Testing Affairs (Dutch: COTAN) evaluated the reliability and construct validity of the test as sufficient [42]. The proportion of items answered correctly per scale was used to compute the scale scores, which were averaged across scales to obtain the total score representing general cognitive ability. These scores were converted to a scale of 0 to 10 for convenience. The estimated reliability of the full test score was α = .87, which was similar to the reliability reported in the test manual [41].

#### Conscientiousness

Conscientiousness was measured using the Dutch version of the Big Five Inventory (BFI; [43]). The entire BFI was administered, but the other scale scores were not used in this study. The conscientiousness scale of the BFI consisted of nine items answered on a five-point Likert scale (1 = strongly disagree through 5 = strongly agree), and Cronbach’s α = .86.

#### Procrastination

Lay's Procrastination Scale [44] was used to measure procrastination tendencies. This scale consisted of 20 Likert items answered on a five-point Likert-scale (1 = never through 5 = all of the time) with Cronbach’s α = .84.

#### Study skills and study habits

The Study Management and Academic Results Test (SMART [45]) consisted of four scales measuring study-related cognitions (academic competence and test competence), and study management skills (time management and strategic studying). Academic competence was defined as being able to understand the study material and enjoying studying the material; test competence was defined as being able to separate essentials from details, managing the amount of study material, coping with tensions, and preparing for examinations; time management was defined as the ability to plan study time and combine studying with leisure activities; and strategic studying was defined as utilizing strategies such as summarizing and testing one’s own knowledge and deliberating how to approach the study material. The SMART contained 29 items, with four to six items per subscale. All items were answered on a four-point Likert scale (1 = almost never through 4 = nearly always). The scales yielded estimated reliabilities of α_academic competence_ = .69, α_test competence_ = .76, α_time management_ = .82, and α_strategic studying_ = .66.

#### Analyses

First, we computed observed correlations and correlations corrected for unreliability in the criterion and the predictors, between the curriculum-sampling test scores and scores on the cognitive and noncognitive measures. Corrections for range restriction could not be applied because the necessary information was not available for the explanatory variables. We expected that the curriculum-sampling tests were saturated with cognitive- and noncognitive constructs, because these constructs also predict academic performance. To check our assumptions about the relationships between the explanatory variables and academic performance, the same analyses were conducted with FYGPA as the dependent variable. The estimate of the reliability of FYGPA in 2015 from Study 1 (see S2 Table) was used in the corrections. Second, we conducted multiple regression analyses with the cognitive and noncognitive predictors described above as independent variables and each of the curriculum-sampling test scores and FYGPA as dependent variables. The *SetCor* function in the psych R package [46] was used to conduct regression analyses based on the correlation matrices corrected for unreliability.

### Results

Descriptive statistics for all variables are in S4 Table. Table 6 shows the correlations between the variables and the multiple regression results after correcting the correlations for unreliability. We will focus on the results after correcting for unreliability, because we were interested in the theoretical relationships between the variables. The results based on the uncorrected correlations are shown in S5 Table. The variance inflation factors (VIF) were all smaller than 4, (the largest VIF before correction was 2.04, and 3.66 after correction, both for the time management variable). Although this value indicates that there were no serious problems with multicollinearity [47] and we adopted a conservative rule of thumb (10 is another often-used threshold, e.g., [48]), some of the results do suggest multicollinearity problems, as we discuss below. This makes some of the results difficult to interpret.

**Table 6:** Construct saturation multiple regression results based on correlations corrected for unreliability.

Variables	Dependent variable					
	Cur. 1		Cur. 2		FYGPA	
	*r*_*c*_	β/*R*^*2*^	*r*_*c*_	β/*R*^*2*^	*r*_*c*_	β/*R*^*2*^
Cognitive ability	.11 [-.08,.30]	.15 (.09)	.10 [-.09,.29]	.17 (.10)	-.13 [-.31,.06]	.01 (.09)
Conscientiousness	.09 [-.10,.27]	.32* (.14)	.18 [-.01,.36]	.58* (.16)	.44* [.27,.58]	.50* (.15)
Procrastination	-.01 [-.20,.18]	.07 (.15)	.03 [-.16,.22]	.31 (.16)	-.32* [-.48,-.14]	.28 (.15)
Academic competence	.24* [.05,.41]	-.28 (.15)	.15 [-.04,.33]	-.08 (.17)	.36* [.18,.52]	-.22 (.16)
Test competence	.49* [.33,.62]	.92* (.14)	.21* [.02,.39]	.47* (.15)	.38* [.20,.53]	.26 (.14)
Time management	.12 [-.07,.31]	-.41* (.15)	-.02 [-.21,.17]	-.39* (.17)	.48* [.32,.62]	.36* (.16)
Strategic studying	.10 [-.09,.29]	.07 (.10)	.08 [-.11,.27]	.09 (.11)	.33* [.15,.49]	.06 (.10)
Model *R*^*2*^ (adj. R^2^)		.38* (.33)		.23* (.17)		.34* (.29)

Cur. 1 = curriculum-sampling test based on literature, Cur. 2 = curriculum-sampling test based on a video lecture, FYGPA = first year mean grade. 95% CI’s are between brackets.

* *p* < .05

In the model with the literature-based curriculum-sampling test as the dependent variable, cognitive ability was not a statistically significant predictor, and the zero-order correlation was also small and not statistically significant. Only conscientiousness, test competence, and time management were significant predictors of the literature-based curriculum-sampling test score in the regression model. However, the independent variables did explain a substantial proportion of the variance in the curriculum-sampling tests scores (Δ*R*^*2*^_*c*_ = .38), and the regression coefficient for the time management score was negative, which contradicts theoretical expectations and the zero-order correlation. These somewhat contradictory results are signs of multicollinearity problems [49]. The model with the video-based curriculum-sampling test scores as the dependent variable showed similar results.

For the model with FYGPA as the dependent variable there was also no statistically significant relationship with the cognitive ability scores. The zero-order correlations for the noncognitive independent variables were all statistically significant and in the expected direction, with conscientiousness and time management as significant predictors in the regression model. Again, procrastination and academic competence switched signs as compared to theoretical expectations and the zero-order correlations. So, the zero-order correlations confirmed our theoretical expectations, but the regression results, again, indicated problems with multicollinearity, which makes them difficult to interpret.

### Discussion

We hypothesized that, similar to academic achievement, the curriculum-sampling test scores were multifaceted compound measures, saturated with cognitive ability and several noncognitive constructs [22,26]. Contrary to our expectations, the scores on the curriculum-sampling tests and first year GPA were not significantly related to cognitive ability as measured by the Q1000 test. One explanation may be restriction in range in the cognitive scores: The students in our sample were a selective group compared to the general population [50]. Another explanation may be that the participants did not put maximum effort in completing the cognitive test because the test was administered low-stakes, which could have impacted the validity of the test scores [51].

We did not find consistent results for the relationships between most noncognitive scale scores and the curriculum-sampling test scores. The unexpected switch of signs for some regression coefficients, and the few statistically significant predictors in the models paired with the relatively high explained variance are indicative of problems with multicollinearity [49], and make it difficult to draw conclusions based on the results. However, the results did indicate a relationship between test competence and the scores on both curriculum-sampling tests. So, we cautiously conclude that there does seem to be some noncognitive saturation in the curriculum-sampling tests, especially of test competence, but the results on the explanatory power of the rest of the noncognitive variables were inconclusive.

## General discussion

The central question in this study was: Do curriculum-sampling test scores predict later academic achievement, and what do they measure in terms of cognitive and noncognitive constructs? The results of the first study showed that the literature-based curriculum-sampling test was a good predictor for short-term and long-term academic achievement. These results were consistent across cohorts and provided an important extension of earlier findings [4].

An interesting and quite remarkable result was that the literature-based curriculum sample test, which is a simple 40-item multiple-choice test, yielded equally good or somewhat better predictions of first year academic achievement than high school GPA (they predicted FYGPA about equally well, but the curriculum-sampling tests was a slightly better predictor for progress and retention in the first year). High school GPA is commonly considered to be the best predictor of academic performance in higher education [11–14] and is a very rich summary measure, containing grades on thoroughly developed national final exams and on secondary school exam performance aggregated over the last three years of high school. Furthermore, the literature-based curriculum-sampling test showed substantial incremental validity over high-school GPA, and a combination of the two explained 37% of the variance in first year GPA and 49% of the variance in third year GPA. In addition, the relationship between the curriculum-sampling test scores and enrollment decisions indicated that curriculum sampling may encourage self-selection and thus may help applicants to gain insight into their fit to the academic program, which can be a considerable advantage.

### Curriculum samples and subject tests

A video-lecture test may have the advantage that the study material is presented in a vivid and attractive format. However, this test explained very little unique variance in addition to the literature-based tests and showed lower predictive validity. In contrast, assessing math skills to predict performance in statistics courses added to the prediction of performance in those courses over the curriculum-sampling test scores. This confirms that assessing specific skills that are needed for distinct components of the curriculum can add to the prediction of performance in those specific components. These findings are in agreement with the content-matching approach of predictors and outcomes [16], including previous findings that scores on the GRE subject tests and other domain-specific graduate school admission tests such as the Medical College Admission Test and the Maths Admission Test, that are more proximal to actual graduate school requirements, were better predictors for graduate school performance than scores on more ‘distal’ tests such as the GRE verbal, quantitative, and analytical tests [17,32]. Similar findings have been reported for the SAT subject tests as compared to SAT I scores [52–54]. However, in these studies, the domain of the subject test was usually not matched to the study domain or major, so we cannot conclude whether these results can also be explained by content-matching. A distinction between subject tests or skills tests and the curriculum sampling approach is that the former aim to assess current knowledge and achievement, while curriculum samples do not; SAT subject tests assess knowledge on high school-level, while curriculum samples require preparation using college-level material. This may or may not matter much in terms of predictive validity, but requiring preparation and assessment on the college level matched to college programs students are applying to, can have benefits when it comes to self-selection and face validity [55]. In addition, for many undergraduate programs, there are no matching high school courses on the basis of which prior achievement can be assessed. That is the case for many theory-oriented programs, but especially applies in the context of assessing nonacademic skills such as communication skills for medical school (e.g.,[56]) or practical skills in vocational education. Note that in these cases, specific skills can also be measured using a samples approach. In future research, it would be interesting to investigate if a samples approach to measuring specific skills would increase their predictive validity.

### Construct saturation

The aim of the second study was to investigate the cognitive and noncognitive construct saturation of the curriculum-sampling tests, in order to explain their predictive validity through possible underlying mechanisms. The results did not completely coincide with our expectations. We did not find relationships between the cognitive ability test scores and the two curriculum-sampling tests scores or first year GPA. On the one hand, this was a surprising finding given the large body of literature that shows a relationship between cognitive abilities and academic performance [32,33], and given the rather obvious cognitive nature of the curriculum-sampling tests. On the other hand, our results are in line with findings based on restricted samples (e.g.,[57]), and with the observation that correlations between cognitive ability and educational achievement tend to decline in every subsequent educational phase due to ongoing selectivity in the educational careers of students [50,58]. In the Netherlands, students are selected for different levels of secondary education around age 12, largely based on a strongly cognitively-loaded test [59]. This degree of early selection restricts the relation between cognitive ability and educational achievement measured in later years (e.g., [60,61]), although small positive relationships between cognitive ability and academic achievement were found in Dutch student samples in earlier studies (e.g., [10,62]). Although our results are based on a relatively small sample and we are, therefore, cautious to draw firm conclusions, our results corresponded to other recent publications that showed that it can be difficult to discriminate between applicants to higher education based on tests of general cognitive skills in restricted or homogeneous samples (e.g., [57]).

Whereas FYGPA was related to and saturated with the noncognitive variables according to expectations based on previous studies [28,34], the relation between curriculum-sampling test scores and the noncognitive variables was not straightforward. Our findings showed that there was some noncognitive saturation in curriculum-sampling tests, mostly for conscientiousness and test competence scores. Thus, this may indicate that the predictive validity of curriculum-sampling tests may partly be explained by its saturation with the competence to prepare for examinations, separating essentials from details, managing the amount of study material, and coping with tensions, which are important learning skills [28,34]. Furthermore, the same rationale applies to the high and robust predictive validity of high school GPA, which can be explained by its multifaceted nature including both cognitive abilities, personality traits, and study skills [63–65].

### Limitations

A first limitation of this study was that the data in each cohort were collected within a single academic program at a single university. Hence, the results do not necessarily generalize to other academic disciplines or to programs focused less on theoretical knowledge and more on practical skills. Future research may extend our study to different programs where different skills may be important, such as communication skills or ethical reasoning (e.g., [66]). A second limitation was that we could not correct for range restriction in calculating the validity of high school GPA, which could lead to an underestimation of their validity. However, since high school GPA was not part of the admission procedure, we expect that the effect of range restriction is small. In addition, the case IV method to correct for indirect range restriction [36] can result in over- or underestimation of the operational validity, although it yields the most accurate estimate of operational correlations compared to other correction methods [67]. Also, we did not differentiate between student who followed the program in English or in Dutch. Investigating possible differential validity for these groups would be an interesting topic for a future study.

Furthermore, we cannot conclude that the positive relationships between the curriculum-sampling test scores and enrollment were caused by the experience of studying for or taking the tests, or by obtaining low scores on the tests. In future studies, a measure of enrollment intentions may be included before the admission tests are administered. In addition, the possibility to promote self-selection through curriculum sampling may also be used in procedures aimed at placement decisions or advising on student-program-fit. In future research it may be investigated whether the predictive validity of curriculum-sampling tests generalizes to lower-stakes procedures.

In study 2, the sample was not entirely representative for the cohort in terms of admission test scores, although the differences were small. A possible explanation for the lower noncognitive saturation of the curriculum-sampling tests as compared to first year GPA is that the curriculum samples were not representative enough for first year courses, which may require more prolonged effort. This is supported by the finding that the grade on the first course in the program was a much better predictor of short-term and long-term academic performance than the scores on the curriculum-sampling tests. Whereas the literature-based curriculum-sampling test mimicked that course, the course required studying an entire book, while the curriculum-sampling test only required studying two chapters. A more comprehensive curriculum sample may be more saturated with constructs related to effortful behavior.

### Conclusions

From existing research we know that high school GPA is a good predictor of future academic performance in higher education and is arguably the most efficient measure to use for admission decisions. In the present study we showed that the literature-based curriculum-sampling tests mostly showed similar or slightly higher predictive validity than high school GPA for first-year academic outcomes, and similar or slightly lower predictive validity for third year academic outcomes. In addition, this curriculum-sampling test showed incremental validity over high school GPA. The hypothesized cognitive- and noncognitive saturation of curriculum samples is often used to explain their high predictive validity. However, our results were difficult to interpret and only showed some noncognitive saturation. Further research is needed to be able to draw conclusions on the construct saturation of curriculum-sampling tests. One caveat of curriculum samples is that the tests should have an acceptable psychometric quality and that extra resources are needed to construct and administer the tests. However, in the format adopted in this study, constructing and administering the tests took relatively little time and effort, and the reliability was sufficient. A final advantage is that curriculum sampling is perceived as a favorable admission method, whereas applicants disliked the use of high school GPA in admission procedures [55]. So, in cases where using high school GPA is not feasible, or when self-selection or applicant perceptions are of major interest, curriculum-sampling tests may be preferred over, or may be used in addition to high school GPA.

## Supporting information

S1 Table Descriptive statistics for predictor variables in Study 1. (PDF)

S2 Table Descriptive statistics for criterion variables in Study 1. (PDF)

S3 Table Observed correlations between predictors and first year academic outcomes per cohort. (PDF)

S4 Table Descriptive statistics for the variables in Study 2. (PDF)

S5 Table Construct saturation multiple regression results based on uncorrected co S1. (PDF)

S1 Appendix Predictive- and incremental validity of curriculum-sampling test scores over high school GPA, based on data of applicants for whom high school GPA data were available. (PDF)

S2 Appendix Incremental validity of specific skills tests over the curriculum-sampling test based on observed correlations. (PDF)
