# Supplementary material for: Admission testing for higher education: A multi-cohort study on the validity of high-fidelity curriculum-sampling tests
Source: PLoS One. 2018 Jun 11;13(6):e0198746. doi: 10.1371/journal.pone.0198746 (PMC5995396; doi:10.1371/journal.pone.0198746)
Supplement: S1 Table — (PDF) [file pone.0198746.s001.pdf]

S1 Table. Descriptive statistics for predictor variables in Study 1.

Variable	n	M_a	M_{r1}	M_{r3}	SD_a	SD_{r1}	SD_{r3}	α_a	α_{r1}	α_{r3}	k
						**2013**					
Cur. 1	851	29.1	29.7	30.7	5.6	5.2	4.6	.81	.77	.73	40
Math	851	16.2	16.6	17.0	4.7	4.7	4.5	.76	.76	.74	30
English	851	13.4	13.7	13.9	3.4	3.3	3.4	.70	.69	.71	20
HSGPA	201		6.7	6.7		0.4	0.5		.73		
FCG	625		6.6	7.0		1.4	1.1		0.74		
						**2014**					
Cur. 1	823	28.9	29.9		5.9	5.4		.82	.80		40
Math	823	17.5	17.8		5.0	5.0		.77	.76		30
English	823	13.7	13.9		2.6	2.5		.58	.55		20
HSGPA	217		6.6			0.5			.77		
FCG	630		6.7			1.6					
						**2015**					
Cur. 1	654	28.7	29.2		5.1	4.7		.76	.74		39
Cur. 2	654	19.1	19.4		3.5	3.3		.71	.69		25
Math	654	17.0	16.9		4.6	4.6		.76	.74		27
HSGPA	118		6.5			0.4			.64		
FCG	515		6.3			1.5					

Note. Cur. 1 = curriculum-sampling test based on literature, Cur. 2 = curriculum-sampling test based on a video lecture, Math = math test, English = English reading comprehension test, HSGPA = high school mean grade, FCG = first course grade, *M* = mean, *SD* = standard deviation, α = Cronbach's alpha, *k* = number of items, $_a$ = based on applicant pool data, $_{r1}$ = based on enrolled student data in year 1, $_{r3}$ = based on enrolled student data in year 3.
