# Supplementary material for: Admission testing for higher education: A multi-cohort study on the validity of high-fidelity curriculum-sampling tests
Source: PLoS One. 2018 Jun 11;13(6):e0198746. doi: 10.1371/journal.pone.0198746 (PMC5995396; doi:10.1371/journal.pone.0198746)
Supplement: S2 Table — (PDF) [file pone.0198746.s002.pdf]

S2 Table. Descriptive statistics for criterion variables in Study 1.

Variable	*n*	*M*	*SD*	$r_{xx'}$
		**2013**		
FYGPA	638	6.6	1.3	.89
FYECT	652	46.0	20.2	
FY dropout[a]	652	0.20		
SGPA	590	6.3	1.8	.74
TGPA	635	6.7	1.2	.88
TYGPA	492	7.1	0.74	.92
TYBA[a]	492	0.53		
		**2014**		
FYGPA	635	6.4	1.4	.88
FYECT	650	44.0	20.1	
FY dropout[a]	650	0.18		
SGPA	610	5.9	1.5	.59
TGPA	633	6.5	1.4	.88
		**2015**		
FYGPA	531	6.6	1.3	.87
FYECT	541	47.0	19.1	
FY dropout[a]	541	0.17		
SGPA	518	6.7	1.5	.65
TGPA	530	6.5	1.2	.85

Note. FYGPA = First year mean grade, FYECT = first year credits, FY dropout = first year dropout, SGPA = statistics courses GPA, TGPA = theoretical courses GPA, TYGPA = third year mean grade, TYBA = third year Bachelor's degree attainment. $r_{xx'}$ = reliability estimate. [a] Proportion.
