# Supplementary material for: Admission testing for higher education: A multi-cohort study on the validity of high-fidelity curriculum-sampling tests
Source: PLoS One. 2018 Jun 11;13(6):e0198746. doi: 10.1371/journal.pone.0198746 (PMC5995396; doi:10.1371/journal.pone.0198746)
Supplement: S3 Table — (PDF) [file pone.0198746.s003.pdf]

S3 Table. Observed correlations between predictors and first year academic outcomes per cohort.

	FYGPA			FYECT			FY dropout[a]			SGPA			TGPA			Enrollment[a]		
Pred.	‘13	‘14	‘15	‘13	‘14	‘15	‘13	‘14	‘15	‘13	‘14	‘15	‘13	‘14	‘15	‘13	‘14	‘15
Cur. 1	.49*	.45*	.44*	.39*	.35*	.33*	-.32*	-.27*	-.20*	.34*	.38*	.35*	.51*	.43*	.44*	.21*	.32*	.22*
	[.43, .55]	[.39, .51]	[.37, .51]	[.32, .45]	[.28, .42]	[.25, .40]	[-.39, -.25]	[-.34, -.20]	[-.28, -.12]	[.27, .41]	[.31, .45]	[.27, .42]	[.45, .57]	[.37, .49]	[.37, .51]	[.15, .27]	[.26, .38]	[.15, .29]
Cur. 2			.29*			.25*			-.13*			.33*			.26*			.18*
			[.21, .37]			[.17, .33]			[-.21, -.05]			[.25, .40]			[.18, .34]			[.11, .25]
Math	.29*	.22*	.25*	.20*	.16*	.18*	-.15*	-.15*	-.07	.34*	.38*	.40*	.25*	.14*	.16*	.15*	.10*	<.01
	[.22, .36]	[.15, .29]	[.17, .33]	[.13, .27]	[.09, .23]	[.10, .26]	[-.22, -.08]	[-.22, -.07]	[-.15, .01]	[.27, .41]	[.31, .45]	[.33, .47]	[.18, .32]	[.06, .22]	[.08, .24]	[.08, .22]	[.03, .17]	[-.08, .07]
English	.25*	.09*		.16*	.08*		-.13*	-.06		.11*	.14*		.27*	.05		.13*	.17*	
	[.18, .32]	[.01, .17]		[.09, .23]	[<.01, .16]		[-.20, -.05]	[-.14, .02]		[.03, .19]	[.06, .22]		[.20, .34]	[-.03, .13]		[.06, .20]	[.10, .24]	
HSGPA	.52*	.47*	.39*	.30*	.30*	.22*	-.22*	-.22*	-.14*									
	[.41, .61]	[.36, .57]	[.26, .50]	[.17, .42]	[.17, .42]	[.08, .35]	[-.35, -.08]	[-.35, -.09]	[-.28, <-.01]									
FCG[b]	.75*	.70*	.68*	.62*	.63*	.57*	-.47*	-.41*	-.40*									
	[.71, .78]	[.66, .74]	[.63, .72]	[.57, .67]	[.58, .67]	[.51, .63]	[-.53, -.41]	[-.47, -.34]	[-.47, -.33]									

Note. Pred. = Predictor, Cur. 1 = curriculum-sampling test based on literature, Cur. 2 = curriculum-sampling test based on a video lecture, Math = math test, English = English reading comprehension test, HSGPA = high school mean grade, FCG = first course grade, FYGPA = first year mean grade, FYECT = first year credits, FY dropout = first year dropout, SGPA = statistics courses GPA, TGPA = theoretical courses GPA, Enrollment = first year enrollment. [a] Point-biserial correlations. [b] For these correlations, results on the first course were not included in the calculation of FYPA and credits. 95% confidence intervals are in brackets. * $p < .05$
