# Supplementary material for: Admission testing for higher education: A multi-cohort study on the validity of high-fidelity curriculum-sampling tests
Source: PLoS One. 2018 Jun 11;13(6):e0198746. doi: 10.1371/journal.pone.0198746 (PMC5995396; doi:10.1371/journal.pone.0198746)
Supplement: S4 Table — (PDF) [file pone.0198746.s004.pdf]

S4 Table. Descriptive statistics for the variables in Study 2.

Variable	*M*	*SD*
Cognitive ability	6.39	1.14
Conscientiousness	3.28	.63
Procrastination	3.17	.48
Academic Competence	3.32	.39
Test Competence	2.99	.46
Time Management	2.88	.62
Strategic Studying	3.02	.51
Cur. 1	28.30	5.18
Cur. 2	20.15	2.96
FYGPA	6.57	.79

Note. Cur. 1 = curriculum-sampling test based on literature, Cur. 2 = curriculum-sampling test based on a video lecture, FYGPA = First year GPA.
