# Supplementary material for: Admission testing for higher education: A multi-cohort study on the validity of high-fidelity curriculum-sampling tests
Source: PLoS One. 2018 Jun 11;13(6):e0198746. doi: 10.1371/journal.pone.0198746 (PMC5995396; doi:10.1371/journal.pone.0198746)
Supplement: S5 Table — (PDF) [file pone.0198746.s005.pdf]

S5 Table. Construct saturation multiple regression results based on uncorrected correlations.

Variables	Dependent variable					
	Cur. 1		Cur. 2		FYGPA	
	r	β/R^2	*r*	β/R^2	*r*	β/R^2
Cognitive ability	.09	.10	.08	.11	-.11	-.02
	[-.10, .28]	(.10)	[-.11, .27]	(.10)	[-.30, .08]	(.09)
Conscientiousness	.07	.13	.14	.30*	.38*	.29*
	[-.12, .26]	(.13)	[-.05, .32]	(.14)	[.20, .53]	(.12)
Procrastination	-.01	.05	.02	.15	-.27*	.06
	[-.20, .18]	(.13)	[-.17, .21]	(.14)	[-.44, -.08]	(.13)
Academic competence	.17	-.02	.10	.03	.28*	<-.01
	[-.02, .35]	(.12)	[-.09, .29]	(.13)	[.09, .45]	(.12)
Test competence	.36*	.44*	.15	.21	.31*	.16
	[.18,.52]	(.12)	[-.04, .33]	(.12)	[.12, .47]	(.11)
Time management	.09	-.16	-.01	-.19	.40*	.21
	[-.10, .28]	(.13)	[-.20, .18]	(.14)	[.22, .55]	(.13)
Strategic studying	.07	.04	.06	.05	.25*	.06
	[-.12, .26]	(.10)	[-.13, .25]	(.11)	[.06, .42]	(.10)
Model R^2 (adj. R^2)		.16*		.09		.24*
		(.10)		(.03)		(.19)

Note. Cur. 1 = curriculum-sampling test based on literature, Cur. 2 = curriculum-sampling test based on a video lecture, FYGPA = first year mean grade. 95% CI’s are between brackets. * $p < .05$
