# Supplementary material for: Admission testing for higher education: A multi-cohort study on the validity of high-fidelity curriculum-sampling tests
Source: PLoS One. 2018 Jun 11;13(6):e0198746. doi: 10.1371/journal.pone.0198746 (PMC5995396; doi:10.1371/journal.pone.0198746)
Supplement: S1 Appendix — (PDF) [file pone.0198746.s006.pdf]

S1 Appendix. Predictive- and incremental validity of curriculum-sampling test scores over high school GPA, based on data of applicants for whom high school GPA data were available.

Observed, true, and operational predictive validity results, aggregated across cohorts.

	FYGPA			FYECT			FY drop out[a]			TYGPA[b]			TYBA[a,b]		
Variable	$\bar{r}$	$\bar{\rho}$	$\bar{r}_c$	$\bar{r}$	$\bar{\rho}$	$\bar{r}_c$	$\bar{r}$	$\bar{\rho}$	$\bar{r}_c$	r	ρ	r_c	r	ρ	r_c
Cur. 1	.43	.58	.52	.33	.44	.39	-.27	-.36	-.32	.40	.66	.59	.17	.30	.27
	[.36,.49]	[.50,.67]	[.44,.60]	[.26,.40]	[.34,.53]	[.31,.47]	[-.34,-.20]	[-.46,-.26]	[-.41,-.23]	[.26,.52]	[.47,.79]	[.42,.71]	[.01,.32]	[.02,.54]	[.02,.48]
HSGPA[c]	.47	.60	.50	.28	.33		-.20	-.24		.61	.76	.65	.30*	.37	.31
	[.41, .53]	[.53,.67]	[.43,.57]	[.20, .35]	[.25,.41]		[-.27, -.12]	[-.32,-.16]		[.50, .70]	[.63,.87]	[.54,.74]	[.15, .44]	[.18,.53]	[.16, .46]

Note. Corrected correlations were obtained using the reliability and range restriction estimates as in the analyses presented in the manuscript. Cur. 1 = curriculum-sampling test based on literature, HSGPA = high school mean grade, FYGPA = first year mean grade, FYECT = first year credits, FY dropout = first year dropout, TYGPA = third year mean grade, TYBA = third year Bachelor's degree attainment, $\bar{r}$ = the aggregated correlation across cohorts, $\bar{\rho}$ = the aggregated true score correlation (corrected for predictor- and criterion unreliability and indirect range restriction), $\bar{r}_c$ = the aggregated operational correlation across cohorts (corrected for indirect range restriction and criterion unreliability). [a] Point-biserial correlations. b Based the 2013 cohort. [c] These correlations could not be corrected for IRR, just for unreliability. 95% confidence intervals are in brackets. All correlations were statistically significant with $p < .05$.

Incremental validity of the literature-based curriculum-sampling test over high school GPA.

	FYGPA			FYECT			TYGPA[a]		
Data	$\bar{R}$	$\bar{R}^2$	$\Delta\bar{R}^2$	$\bar{R}$	$\bar{R}^2$	$\Delta\bar{R}^2$	R	R^2	ΔR^2
Observed	.52	.27	.05	.36	.13	.05	.62	.39	.01
Corrected	.58	.34	.09	.40	.16	.08	.71	.50	.08

Note. FYGPA = first year mean grade, FYECT = first year credits, TYGPA= third year mean grade, $\bar{R}$ = aggregated multiple correlation, $\bar{R}^2$ = aggregated variance explained based on HSGPA and the curriculum-sampling test scores, ΔR^2 = aggregated increase in explained variance based on curriculum-sampling test scores over HSGPA. Observed = based on observed correlations, Corrected = based on operational correlations (corrected for IRR).
[a] Based on the 2013 cohort.
