# Supplementary material for: Admission testing for higher education: A multi-cohort study on the validity of high-fidelity curriculum-sampling tests
Source: PLoS One. 2018 Jun 11;13(6):e0198746. doi: 10.1371/journal.pone.0198746 (PMC5995396; doi:10.1371/journal.pone.0198746)
Supplement: S2 Appendix — (PDF) [file pone.0198746.s007.pdf]

S1 Appendix. Incremental validity of specific skills tests over the curriculum-sampling test based on observed correlations.

The incremental validity of the math test over the curriculum-sampling test for SGPA based on the observed correlations equaled $\Delta\bar{R}^2$ = .08 (R^2 = .21). The aggregated correlation between the math test scores and the curriculum-sampling test scores was $\bar{r}_c$ = .27. The incremental validity of the English test over the curriculum-sampling test for TGPA based on the observed correlations equaled $\Delta\bar{R}^2$ = < .01 (R^2 = .21). The aggregated correlation between scores on the English test and scores on the curriculum-sampling test was $\bar{r}_c$ = .44. When the analyses were conducted for each cohort separately (results not shown but available upon request), the incremental validity of the math test over the literature-based curriculum-sampling test for statistics GPA was statistically significant in each cohort. The incremental validity of the English test over the literature-based test was only statically significant in the 2013 cohort.
